# Effects of naringin on physical fatigue and serum MMP-9 concentration in female rats

**DOI:** 10.1080/13880209.2016.1244553

**Published:** 2016-12-09

**Authors:** Mohammad Zamanian, Mohammadreza Hajizadeh, Ali Shamsizadeh, Mohammad Moemenzadeh, Morteza Amirteimouri, Mohammad Elshiekh, Mohammad Allahtavakoli

**Affiliations:** a Physiology-Pharmacology Research Center and Department of Physiology, Rafsanjan University of Medical Sciences, Rafsanjan, Iran;; b Department of Clinical Biochemistry, Rafsanjani University of Medical Sciences, Rafsanjan, Iran;; c Molecular Medicine Research Center, Rafsanjan University of Medical Sciences, Rafsanjan, Iran;; d Department of Physiology Faculty of Medicine, University of Medical Sciences, Tehran, Iran

**Keywords:** Lactate dehydrogenase, glucose, exhaustive swimming test

## Abstract

**Context:** Oxidative stress has a critical role in the development of physical fatigue and activation of matrix metalloproteinases-9 (MMP-9). Naringin (Nar) is a bioflavonoid that has antioxidant activity and suppresses MMP-9 expression.

**Objective:** The present study evaluates the anti-fatigue activity of Nar on physical fatigue and serum MMP-9 concentration in rats.

**Materials and methods:** Fifty female Wistar rats were randomly divided into five groups (*n* = 10); a control group, vehicle group and three Nar treatment groups. The Nar treated groups received different doses of Nar (40, 80 and 160 mg/kg/day) for 30 days. On the 30^th^ day, rats were sacrificed immediately after exhaustive swimming test. Serum MMP-9 concentration and several biochemical parameters related to fatigue were measured.

**Results:** Exhaustive swimming time in the Nar-80 group significantly increased 1.78-, 1.53-, 1.5- and 1.3-fold compared with the control, vehicle, Nar-40 and Nar-160 groups, respectively. In addition, exhaustive swimming time in the Nar-160 group significantly increased 1.36-fold compared with the control group. Nar-80 significantly decreased LDH activity by 60.45% and 57.47% compared with the vehicle and control groups, respectively. Furthermore, Nar-80 and Nar-160 increased blood glucose levels by 19.56% and 18.38% compared with the control group, respectively. Nar-80 and Nar-160 significantly decreased serum MMP-9 concentration by 61.57% and 83.39% compared with the control group, respectively.

**Conclusion:** Based on our data, Nar has anti-fatigue effects which may be attributed to its property in modulating energy metabolism and reducing serum MMP-9 concentration. Thus, Nar may be a promising agent for the treatment of physical fatigue.

## Introduction

Physical fatigue is a complex physiological process that happens with physical stress or strenuous exercise, which reduces exercise endurance (Mehta & Agnew 2012; Kumar et al. 2013). There are several theories about mechanisms of fatigue resulting from physical exercise which include ‘exhaustion theory’ (reducing energy sources such as glucose along with liver glycogen), ‘clogging theory’ (accumulation of serum lactic acids and blood urea nitrogen) and ‘radical theory’ (free radical generation) (You et al. 2011). Intensive exercise, as well as exhaustive exercise, can elevate oxidative stress, leading to an imbalance between the body’s oxidation system and antioxidant enzymes. Hence, accumulation of free radicals such as reactive oxygen species (ROS) can cause damage to many parts of the cells such as proteins, DNA, and cell membranes by stealing their electrons via a process called oxidation, which leads to decreases in performance and fatigue (Powers et al. 2011; Lin et al. 2014).

Matrix metalloproteinase 9 (MMP-9), or 92-kD type IV collagenase is a zinc-dependent peptidase that belongs to MMPs family and is expressed by many cells, including neurons, skeletal muscle cell and endothelial cells (Wang et al. 2013). It has been reported that MMP-9 mediates a crucial role in cleaving muscle-specific proteins and participating in the extracellular matrix formation, remodelling and regeneration in skeletal muscle (Urso et al. 2009). It has been shown that a mild exercise could induce MMP-9 expression in skeletal muscle (Rullman et al. 2009). Previous studies demonstrated that after physical exercise, serum MMP-9 concentration and MMP-9 mRNA expression in skeletal muscle increased (Rullman et al. 2007; Reihmane et al. 2012). It has been also suggested that increased oxidative stress leads to up-regulation of MMP-9 expression (Kameda et al. 2003).

Several experimental studies in animals have shown that exogenous nutritional supplementation such as bioflavonoids scavenge free radicals and enhance endurance physical exercise performance through postponing muscle fatigue (Lin et al. 2014; Su et al. 2014). Naringin (4′, 5, 7-trihydroxyflavanone-7-rhamnoglucoside) (Nar) is a bioflavonoid with eight free aromatic hydroxyl groups that is extracted from grapefruit and citrus species (Kandhare et al. 2014). Recent studies have reported that Nar possess biologically and pharmacologically broad spectrum antioxidant (Bacanlı et al. 2015) and anti-inflammatory (Chtourou et al. 2016) and hepatoprotective (Pari & Amudha 2011) properties. It has been recently demonstrated that Nar in an *in vitro* significantly suppresses the overexpression of MMP-9 (Aroui et al. 2016b).

To the best of our knowledge, no study has reported the Nar anti-fatigue properties. In this study evaluates effects of Nar on physical fatigue and serum matrix metalloproteinase-9 concentration in female rats using exhaustive swimming test.

## Materials and methods

### Animals

Fifty female Wistar rats (9 weeks old, 1 8 0–220 g) were obtained from physiology-pharmacology research Center of Rafsanjan University of Medical Sciences, Iran. The rats were housed in standard cages under automatically controlled air conditions, temperature (24 ± 2 °C) and humidity (60%) with a 12 h light/dark cycle. Animals had access to food and water *ad libitum*. Experiments were performed according to the Guide for the Care and Use of Laboratory Animals (Institute for Laboratory Animal Research, National Research Council, Washington, DC, National Academy Press, no. 85-23, revised 1996). Experimental protocol in our present study was approved by Animal Ethics Committee of Rafsanjan University of Medical Sciences (Approval ID: IR.RUMS.REC.1394.121).

### Experimental design

After acclimatization period of at least 7 days, rats (*n* = 50) were randomly divided into five equal groups (*n* = 10): (1) control group; (2) vehicle group (V); (3) Nar-40 group (40 mg/kg); (4) Nar-80 group (80 mg/kg) and (5) Nar-160 group (160 mg/kg). Nar with purity of ≥95% was purchased from Sigma Chemical Co. (St. Louis, MO). The doses of Nar were selected based on the preliminary experiments in our laboratory and previous studies (Kandhare et al. 2012, 2014).

Distilled water (3.0 mL/kg BW/day) was orally administered to vehicle group; while other groups were treated with the corresponding same volume of Nar (40, 80 and 160 mg/kg BW/day in distilled water) for 30 days.

### Measurement of the weight-loaded swimming capacity

Rats were allowed to swim without loading for 15 min three times per week to customize them to swimming. On the 30^th^ day of the experiment, rats were taken for weight-loaded swimming test. The procedures used in this experiment as previously described by Chang et al. (2013). Briefly, 30 min after the last treatment, the animals were dropped separately into a columnar swimming pool (65 cm tall and radius 20 cm) filled with fresh water maintained at 27 ± 1 °C, approximately 40 cm deep so that rats could not support themselves by touching the bottom with their tails. A (steel ring) weighting equivalent to 5% of body weight was loaded on the tail root of each rat. The swimming time to exhaustion was used as the index of the forced swimming capacity. The animal exhaustion time was recorded when they failed to rise to the surface of the water to breathe within 7 s.

### Sample preparation

Immediately after the exhaustive swimming exercise, all animals were sacrificed by decapitation for biochemical analyses. Blood samples were collected in the tubes without anticoagulant. The blood samples for serum analyses were centrifuged at 3000 rpm for 10 min at 4 °C.

### Measurement of biochemical parameters related to fatigue

Using an autoanalyzer (Biotecnica, BT 4500, and Rome, Italy), the levels of glucose, blood urea nitrogen (BUN), creatinine, uric acid, total cholesterol (TC), triglyceride (TG), direct bilirubin (DBIL), total bilirubin (TBIL), albumin and lactate dehydrogenase (LDH), creatine kinase (CK), alanine aminotransferase (ALT), aspartate aminotransferase (AST) and alkaline phosphatase (ALP) activity in the serum were determined.

### Determination of serum MMP-9 concentration

The serum concentration of MMP-9 was determined using enzyme-linked immunosorbent assay (ELISA) (R&D systems, Minneapolis, MN) according to the manufacturer’s instructions. Briefly, 50 μL of assay diluents was added to 50 μL of the samples and standards were added to the ELISA plate and incubated for 2 h at room temperature. After removing the remained serum with washing solution, 100 μL of HRP-conjugated secondary antibody was added and after 2 h incubation was washed. In the next step, 100 μL of substrate (TMB + H_2_O_2_) was added and after 30 min the stop solution (H_2_O_2 _+_ _2 N) was added and the optical density of each sample was measured using an ELISA reader instrument (Bio-Rad, Hercules, CA) at 450 nm.

### Statistical analysis

Data are expressed as mean ± S.E.M and were analyzed by one-way ANOVA. Individual differences were determined by Tukey’s test. A value of *p* < 0.05 was considered significant.

## Results

### Effect on body weights of rats

The data of body weights were recorded before the experiment and on the 30^th^ day. As shown in Table 1, there no significant changes in the initial and final body weight among control, vehicle, Nar-40, Nar-80 and Nar-160 groups.

**Table 1.** Effect of Nar on body weights of rats.

Groups	Initial (g)	Final (g)
Control	201 ± 5.17	205 ± 4.43
Vehicle	207.75 ± 8.52	214.87 ± 7.15
Nar-40	201.85 ± 8.4	204.85 ± 8.47
Nar-80	196.85 ± 5.97	200.14 ± 7.4
Nar-160	204.71 ± 6.53	207.71 ± 8.73

Data are expressed as mean ± S.E.M (*n* = 10).

### Effect of Nar on exhaustion swimming time

**Figure 1.**
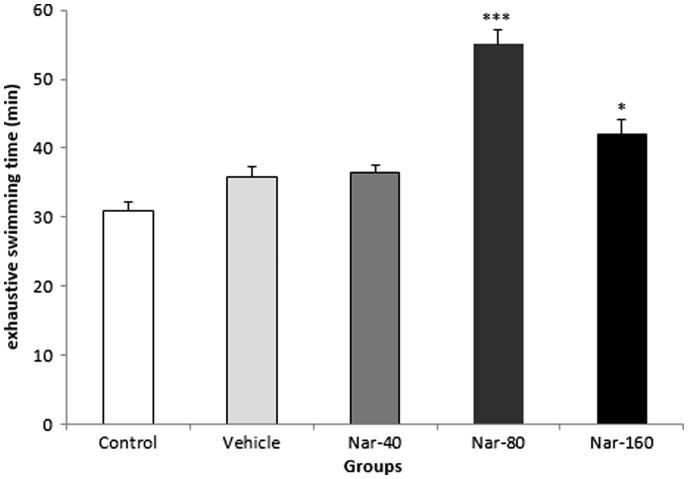
Effect of Nar on swimming time to exhaustion. Data are expressed as mean ± SEM, (*n* = 10). ***p < 0.001 when compared with other groups and *p < 0.05 Control group.

As shown in Figure 1, the exhaustion swimming time of rats treated with Nar-80 showed a significant difference compared with the control group and other groups (*p* < 0.001). The rats treated with Nar-80 showed approximately 1.78-, 1.53-, 1.5- and 1.3-fold increases in the exhaustion swimming time when compared with the control, vehicle, Nar-40 and Nar-160 groups, respectively. In addition, compared with Control group rats treated with Nar-160 showed approximately 1.36-fold increases in the exhaustion swimming time (*p* < 0.05).

### Effect of Nar on serum biochemical parameters in rats

A summary of biochemical parameter results is presented in Table 2.

**Table 2.** Effect of Nar on serum biochemical parameters.

Parameters control	Vehicle	Nar-40	Nar-80	Nar-160
AST (U/L) 186 ± 16	181 ± 17	170 ± 26	227 ± 31	208 ± 14
ALT (U/L) 116 ± 11	113 ± 11	113 ± 7	103 ± 20	101 ± 28
ALP (U/L) 384 ± 48	387 ± 50	431 ± 40	317 ± 36	388 ± 34
CK (U/L) 3009 ± 318	3131 ± 469	2440 ± 598	2557 ± 666	3035 ± 570
LDH (U/L) 2253 ± 171	2095 ± 135	1797 ± 128	891 ± 35^a^	1955 ± 175
Urea (mg/dL) 56 ± 1	54 ± 1	55 ± 3	46 ± 3	51 ± 1
Glucose (mg/dL) 111 ± 5	112 ± 2	126 ± 7	138 ± 3^b^	136 ± 3^b^
Albumin (g/dL) 3.8 ± 0.0	3.8 ± 0.0	3.9 ± 0.1	4 ± 0.0	3.8 ± 0.0
TBIL (μg/dL) 273 ± 2 0	262 ± 18	214 ± 14	257 ± 20	217 ± 37
DBIL (μg/dL) 107 ± 4	112 ± 12	100 ± 0.0	128 ± 18	100 ± 0.0
BUN (mg/dL) 26 ± 0.7	25 ± 0.7	25 ± 1	21 ± 1	23 ± 1
CR (mg/dL) 0.64 ± 0.03	0.66 ± 0.02	0.71 ± 0.02	0.65 ± 0.02	0.62 ± 0.02
UA (mg/dL) 2 ± 0.3	1.91 ± 0.11	1.75 ± 0.05	1.87 ± 0.13	1.77 ± 0.12
TG (mg/dL) 75 ± 9	71 ± 10	67 ± 6	75 ± 12	84 ± 15
TC (mg/dL) 87 ± 4	82 ± 3	78 ± 2	91 ± 5	79 ± 4

Data are expressed as mean ± SEM (*n* = 10).

AST: aspartate aminotransferase; ALT: alanine aminotransferase; ALP: alkaline phosphatase; CK: creatine kinase; TBIL: total bilirubin; DBIL: direct bilirubin; BUN: blood urea nitrogen; CR: Creatinine; UA: uric acid; TG: triacylglycerol; TC: total cholesterol.

a *p* < 0.001 compared with other groups.

b *p* < 0.01 compared with control and vehicle groups.

Compared with other groups, rats treated with Nar-80 showed a significant decrease in LDH activity (*p* < 0.001). In addition, compared with control and vehicle groups, animals that treated with Nar-80 (*p* < 0.01) and Nar-160 (*p* < 0.01) showed a significant increase in blood glucose levels. There were no statistically significant differences in other biochemical parameters among all groups.

### Effect of Nar on serum MMP-9 concentration

**Figure 2.**
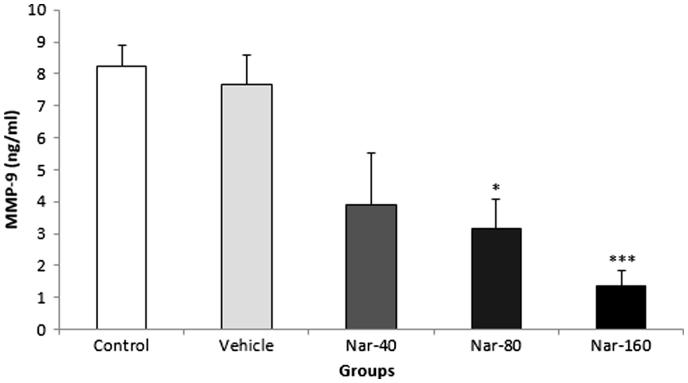
Effect of Nar on serum MMP-9 concentration. Data are expressed as mean ± SEM (*n* = 10). *p < 0.05; ***p < 0.001 when compared with the control and vehicle groups.

As shown in Figure 2, compared with control and vehicle groups rats treated with Nar-80 and Nar-160 showed a significant decrease in the serum MMP-9 concentration (*p* < 0.05) and (*p* < 0.001) respectively. Nar-80 and Nar-160 significantly decreased serum MMP-9 concentration by 61.57% and 83.39% compared with the control group, respectively. Furthermore, Nar-80 and Nar-160 significantly decreased serum MMP-9 concentration by 58.72% and 82.16% compared with the vehicle group, respectively.

## Discussion

This study evaluates effects of Nar on physical fatigue and serum MMP-9 concentration. In our current study, we demonstrated that administration of Nar-80 significantly extended the weight-loaded swimming time to exhaustion, improved swimming endurance capacity and reduced serum MMP-9 concentration.

Exercise-induced physical fatigue can be evaluated by blood and muscular parameters that are associated with energy metabolism, including blood glucose, urea nitrogen, and lactic acid, as well as muscular glycogen. It is well recognized that muscular exercise results in rapid ATP consumption, and energy deficiency, which is a critical precipitating factor to physical fatigue (Huang et al. 2011). In the present study, we selected a weight-loaded forced swimming that is a common experimental exercise model to evaluate the effectiveness of physical fatigue and the maximum swimming time is directly related to the degree of fatigue (Chang et al. 2013). In addition, our results showed that exhaustion swimming times significantly prolonged in rats treated with Nar-80 (80 mg/kg) compared with the control and other treatment groups.

The homeostasis of blood glucose, a breakdown product of skeletal and liver glycogen, plays an essential role during prolong and strenuous exercise (Wu et al. 2013). Exhaustive exercise usually results in hypoglycemia, which can suppress brain activity; hence, the speed and degree of fatigue development can explain by blood glucose levels (Kumar et al. 2013). Therefore, glucose is an acceptable index to evaluate fatigue. In our present study, we demonstrated that rat treated with high dose of Nar (Nar-80 and Nar-160) showed a significant increase in the blood glucose levels compared with the control and vehicle group. However, the increase in blood glucose levels may be one pathway of Nar to mediated anti-fatigue effect.

Strenuous exhaustive exercise can produce an imbalance between the reactive oxygen species (ROS) and antioxidant enzymes and thereby generating more ROS can damage muscle cellular components and leads to fatigue (Ding et al. 2011; Lin et al. 2014). Serum lactate dehydrogenase (LDH) activity is another important marker of muscle damage during strenuous exercise (Xu et al. 2013), as it catalyzes the reversible transformation of pyruvate to lactate, with the accompaniment oxidation of NADH to NAD^+^. During anaerobic conditions, LDH becomes the main enzyme because of its ability to regenerate NAD^+ ^and permitting continued carbon flow via the glycolytic pathway to support ATP synthesis (Elustondo et al. 2013). Our data showed that Nar-80 produced a significant decrease in LDH activity compared with the other groups. The lower LDH activity in Nar-80 group suggested that Nar may have an ability to attenuate muscle damage during strenuous swimming.

It has been reported that strenuous exercise induces MMP-9 levels (Koskinen et al. 2002; Rullman et al. 2007, 2009). Furthermore, many studies have suggested that increased oxidative stress leads to up-regulation of MMP-9 expression (Uemura et al. 2001; Kameda et al. 2003) as well as free radicals such as ROS can be activated MMP-9 expression (Rajagopalan et al. 1996). Uemura et al. (2001) indicated that antioxidants therapy can reduce MMP-9 activity through reducing oxidative stress. Fast glycolytic fibres have more vulnerability to fatigue than slow oxidative muscle fibres (type I fibre) because it has low content and activity of oxidative enzymes than slow muscle fibres (type I fibre) (Westerblad et al. 2010; Bogdanis 2012). The increased levels of MMP-9 in the skeletal muscle of mice can change slow muscle fibres (type I fibre) into fast glycolytic fibres (type II fibre) (Dahiya et al. 2011). Therefore, MMP-9 may be a new marker related to muscle fatigue.

Recent studies demonstrated that Nar can scavenge free radicals and can reduce oxidative stress via increases in antioxidant enzymes activity such as superoxide dismutase (SOD) and glutathione peroxidase (GPx) (Bacanlı et al. 2015; Rajadurai & Stanely Mainzen Prince 2006; Chtourou et al. 2015). Nar treatment reduced the enzymatic activity and protein level of MMP-9 in U87cells (Aroui et al. 2016a). Aroui et al. (2016b) showed that different concentrations of Nar decreased the level of MMP-9 expression in U251 glioma cells. Lee et al. (2009) reported that Nar treatment suppressed MMP-9 expression via the transcription factors NF-κB and activator protein-1 in TNF-α-induced vascular smooth muscle cells. In our present study, we showed that administration of Nar treated with difference doses 80 and 160 mg/kg produced significant reduce in serum MMP-9.

Our present study showed that Nar-80 administration resulted in substantially prolonged exhaustion swimming times, increased blood glucose levels, decreased LDH activity and reduced serum MMP-9. In addition, Nar-160 administration produced substantial elevate blood glucose levels and reduced serum MMP-9. These results indicate that Nar possesses anti-fatigue activity, which may be mediated via increased energy sources as shown by increasing blood glucose levels, reducing serum MMP-9 concentration and the LDH activity as a marker of muscular damage, as well as may be through inhibited free radicals generation.

## Conclusions

Our current study, demonstrate that the administration of Nar for 30 days 80 mg/kg resulted in increased exhaustion swimming times and reduced MMP-9 concentration in female rats. In addition, Nar with dose 80 mg/kg led to increasing of blood glucose levels and reducing LDH activity. These results indicated that Nar with dose 80 mg/kg has anti-fatigue activity. This activity may be mediated by reducing serum MMP-9 concentration, reducing LDH activity and increasing blood glucose levels as energy sources. More studies are needed to confirm that Nar may act as a therapeutic agent for treatment of physical fatigue.
